# Impact of Race and Ethnicity on Outcomes After Mitral Transcatheter Edge-to-Edge Repair: Analysis From the COAPT Trial

**DOI:** 10.1016/j.jscai.2025.103822

**Published:** 2025-10-30

**Authors:** Mahesh V. Madhavan, Oludamilola Akinmolayemi, Oluseun O. Alli, Shmuel Chen, D. Edmund Anstey, Bjorn Redfors, Bahira Shahim, Janani Aiyer, Yu Shu, William T. Abraham, JoAnn Lindenfeld, Michael J. Mack, Gregg W. Stone

**Affiliations:** a Columbia Unversity Irving Medical Center and the NewYork-Presbyterian Hospital, New York, New York; b Cardiovascular Research Foundation, New York, New York; c Mount Sinai Fuster Heart Hospital, Icahn School of Medicine at Mount Sinai, New York, New York; d Novant Health Heart and Vascular Institute—Elizabeth, Charlotte, North Carolina; e Weill Cornell Medical Center and the NewYork-Presbyterian Hospital, New York, New York; f University of Gothenburg, Gothenburg, Sweden; g Department of Medicine, Karolinska Institute, Stockholm, Sweden; h Abbott Laboratories, Santa Clara, California; i The Ohio State University Wexner Medical Center, Columbus, Ohio; j Vanderbilt University Medical Center, Nashville, Tennessee; k Baylor Scott and White Heart Hospital Plano, Plano, Texas

**Keywords:** secondary mitral regurgitation, transcatheter edge-to edge repair, race, ethnicity

## Abstract

**Background:**

Few studies have evaluated race and ethnicity-based differences in outcomes in patients with heart failure and secondary mitral regurgitation (SMR). We examine the impact of race and ethnicity on outcomes after treatment of severe SMR with mitral transcatheter edge-to-edge repair (M-TEER) with the MitraClip device plus guideline-directed medical therapy (GDMT) compared with GDMT alone in the Cardiovascular Outcomes Assessment of the MitraClip Percutaneous Therapy for Heart Failure Patients with Functional Mitral Regurgitation (COAPT) trial.

**Methods:**

Patients in the COAPT trial were stratified by self-identified race and ethnicity as White, Black, or Hispanic. Outcomes assessed included echocardiographic core laboratory-assessed mitral regurgitation (MR) reduction, the composite outcome of all-cause mortality or heart failure hospitalization (HFH), and change in quality of life as measured by the Kansas City Cardiomyopathy Questionnaire (KCCQ) during 2-year follow-up.

**Results:**

Of 585 patients, 457 (78.1%) identified as White, 88 (15.0%) identified as Black, and 40 (6.8%) identified as Hispanic. At 2 years, MR reduction was present with M-TEER in White and Black patients, and KCCQ improvement was observed in White and Hispanic patients. The 2-year rate of mortality or HFH with M-TEER compared with GDMT alone was lower in White patients (hazard ratio [HR], 0.59; 95% CI, 0.46-0.75) and Black patients (HR, 0.30; 95% CI, 0.14-0.61), but not Hispanic patients (HR, 1.09; 95% CI, 0.46-2.57; *P*_interaction_ = .06).

**Conclusions:**

In the COAPT trial, MitraClip treatment reduced MR in White and Black patients and improved quality of life in White and Hispanic groups. Freedom from all-cause mortality or HFH through 2-year follow-up improved with M-TEER compared with GDMT alone in White patients and possibly to an even greater extent in Black patients. The small number of Hispanic patients enrolled precludes definitive conclusions in that group.

## Introduction

The varying prevalence of cardiovascular diseases and clinical outcomes among racial and ethnic groups have been previously described.^1^ Minority group patients in the United States are often unrepresented in cardiovascular studies and clinical trials, contributing to a lack of knowledge regarding the natural history and optimal treatment strategies for these patient cohorts.^2^ Previous analyses focused on structural heart interventions have suggested that racial disparities concerning utilization, outcomes, and health care costs persist despite improvements in the contemporary era.^3^^,^^4^ However, there is a paucity of high-quality data to inform treatment with mitral transcatheter edge-to-edge repair (M-TEER) in patients belonging to minority groups.^5^, ^6^, ^7^

The Cardiovascular Outcomes Assessment of the MitraClip Percutaneous Therapy for Heart Failure Patients with Functional Mitral Regurgitation (COAPT) trial demonstrated that treatment of moderate-to-severe (3+) or severe (4+) secondary mitral regurgitation (SMR) with the MitraClip device (Abbott) plus guideline-directed medical therapy (GDMT) was safe and effective in improving outcomes in patients with heart failure (HF) compared with GDMT alone.^8^^,^^9^ However, outcomes by race or ethnicity have not been reported. This analysis assessed the impact of race and ethnicity on outcomes of patients with HF and severe SMR treated with M-TEER plus GDMT compared with GDMT alone in the COAPT trial.

## Materials and methods

### Study design and patient population

Detailed description of the COAPT trial design, protocol, and principal results have been previously published.^8^, ^9^, ^10^ Briefly, COAPT was a multicenter, randomized, controlled, parallel-group trial that evaluated the effectiveness and safety of M-TEER with the MitraClip system in patients with symptomatic HF and moderate-to-severe or severe SMR. The trial was registered on ClinicalTrials.gov (NCT01626079).

COAPT enrolled patients with moderate-to-severe (3+) or severe (4+) SMR with ischemic or nonischemic cardiomyopathy, left ventricular ejection fraction (LVEF) 20% to 50% and who were symptomatic (New York Heart Association class ≥ II) despite maximally tolerated doses of GDMT. Key exclusion criteria were left ventricular end-systolic dimension of >70.0 mm, severe pulmonary hypertension, and moderate or severe right ventricular dysfunction. Enrolled patients were randomized to undergo M-TEER with the MitraClip plus GDMT or to receive GDMT alone. The trial was approved by the institutional review board at all participating centers, and all enrolled patients provided written informed consent.

The provision of race and ethnicity data from patients was voluntary and was asked as a single question based on self-identification. The categories offered to participants to choose from were White or Caucasian, Black or African American, Hispanic or Latino, Asian, Native Hawaiian or Other Pacific Islander, American Indian or Alaska Native, and Other. For this study, patients were classified as White or Caucasian (White), Black or African-American (Black), or Hispanic or Latino (Hispanic). Other groups were excluded from this analysis given their limited enrollment in the COAPT trial.

Routine clinical and echocardiographic follow-up was performed through 5 years from randomization. At 2 years, crossover treatment with MitraClip therapy was allowed in GDMT alone group patients who still met all qualifying entry criteria.

### Clinical end points

The primary effectiveness outcome for the present study was the composite of all-cause mortality or heart failure hospitalization (HFH) through 2-year follow-up, before allowed crossover treatment with the MitraClip in the GDMT alone group. Secondary outcomes included the components of the primary outcome, change in MR severity, and quality of life (QoL) as assessed by the Kansas City Cardiomyopathy Questionnaire (KCCQ) score. Sensitivity analyses were performed at 5 years. The primary safety outcome for the present study was the occurrence of device-specific events (single-leaflet device attachment), device embolization, endocarditis requiring surgery, mitral stenosis requiring surgery, or any device-related complication requiring nonelective cardiovascular surgery) or progressive HF unrelated to device complications (left ventricular assist device or heart transplantation) at 30 days.

An independent committee adjudicated clinical outcomes according to prespecified definitions after review of original source documents. An independent echocardiographic core laboratory assessed the severity of MR and other echocardiographic parameters according to the American Society of Echocardiography criteria at baseline and follow-up assessment.

### Statistical analysis

Descriptive statistics for clinical and echocardiographic characteristics by race and ethnicity at baseline are presented as proportions for categorical variables and means with standard deviations (SD) for continuous variables. Differences in patient characteristics between groups were compared using the χ^2^ test or Fisher exact test for categorical variables and linear mixed-effect model for continuous variables. Comparisons across 3 groups were performed with analysis of variance for continuous variables and χ^2^ test or Fisher exact test for categorical variables.

All effectiveness analyses were performed in the intention-to-treat population. Safety outcomes in the M-TEER group were assessed in patients in whom MitraClip implantation was attempted. For other outcomes, time-to-first event rates between groups were estimated with Kaplan-Meier analysis and compared with Cox proportional hazards regression analysis. Relative rates are presented as hazard ratios (HR) and 95% confidence intervals (CI). Interactions between race and ethnicity, randomization to the MitraClip device, and the rates of outcomes were assessed using Cox proportional hazards model. The impact of race and ethnicity was determined in a multivariable model created using stepwise regression in which variables were entered into the model at the 0.2 significance level and removed at the 0.1 level (from the Wald χ^2^ statistic). Variables were eligible for inclusion if present for 90% of patients with no cell having <30 patients. Variables entered in the final model included baseline use of renin angiotensin system antagonists, aldosterone inhibitors, vasodilators (hydralazine or nitrates), β blockers, a history of atrial fibrillation, anemia, and renal disease, body surface area, chronic obstructive pulmonary disease, serum creatinine, diabetes, diastolic blood pressure, effective regurgitant orifice area (EROA), sex, KCCQ overall summary score, LVEF, previous percutaneous coronary intervention or coronary artery bypass graft, peripheral vascular disease, B-type natriuretic peptide or N-terminal B-type natriuretic peptide, 6-minute walk distance, previous stroke, Society of Thoracic Surgeons (STS) replacement score, tricuspid regurgitation grade, race and ethnicity, and randomized treatment group. Two-sided *P* values <.05 were considered statistically significant. All statistical analyses were performed using SAS software, version 9.4 (SAS Institute).

## Results

### Study population and baseline characteristics

A total of 614 patients from 78 centers were enrolled in the COAPT trial, all of whom voluntarily provided self-identified data on race and ethnicity. A total of 457 patients (74.4%) were White, 88 (14.3%) were Black, 40 (6.5%) were Hispanic, and 29 patients (4.7%) did not fall into any of these groups. The distribution of all race and ethnicity categories included in COAPT trial and their respective prevalence in the United States are provided in Supplemental Table S1. Among the 585 patients in this analysis population, 78.1%, 15.0%, and 6.8% of patients were White, Black, and Hispanic, respectively.

Baseline clinical characteristics by race and ethnicity pooled across treatments and separately by treatment arm are presented in Table 1 and Supplemental Table S2, respectively. Baseline medications at the time of enrollment by race and ethnicity are provided in Supplemental Table S3. White patients were older, more often male, and had a higher prevalence of comorbidities and a higher baseline STS score for mitral valve repair and replacement. However, diabetes was least frequently present in White patients. Black patients were more often women and were more likely to present with nonischemic cardiomyopathy compared with White and Hispanic patients. Hispanic patients were the least symptomatic at baseline according to baseline KCCQ score. Black patients were more likely to report taking nitrates and hydralazine at baseline compared with White and Hispanic patients.

**Table 1:** Baseline clinical and echocardiographic characteristics by race and ethnicity.

Characteristic	White (n = 457)	Black (n = 88)	Hispanic (n = 40)	*P*
Age, y	74.0 ± 9.9	65.4 ± 12.3	68.4 ± 15.0	<.0001
Male sex	303 (66.3)	39 (44.3)	29 (72.5)	.0002
Diabetes mellitus	155 (33.9)	41 (46.6)	19 (47.5)	.03
Hypertension	367 (80.3)	75 (85.2)	31 (77.5)	.48
Hypercholesterolemia	259 (56.7)	38 (43.2)	17 (42.5)	.02
Peripheral vascular disease	96 (21.0)	11 (12.5)	1 (2.5)	.005
History of anemia	98 (21.4)	28 (31.8)	12 (30.0)	.07
History of atrial fibrillation or flutter	269 (58.9)	40 (45.5)	13 (32.5)	.001
Coronary artery disease	354 (77.5)	45 (51.1)	28 (70.0)	<.0001
Prior myocardial infarction	252 (55.1)	32 (36.4)	17 (42.5)	.003
Prior PCI	230 (50.3)	23 (26.1)	15 (37.5)	<.0001
Prior CABG	209 (45.7)	15 (17.0)	14 (35.0)	<.0001
Prior stroke or TIA	70 (15.3)	23 (26.1)	8 (20.0)	.04
COPD	111 (24.3)	25 (28.4)	2 (5.0)	.01
STS risk score for replacement	8.7 ± 6.0	6.2 ± 4.4	6.9 ± 5.8	.0003
≥8%	210 (46.0)	29 (33.0)	13 (32.5)	.03
STS risk score for repair	6.2 ± 5.3	4.5 ± 6.6	4.4 ± 4.4	.007
≥8%	115 (25.2)	11 (12.5)	5 (12.5)	.01
Body mass index, kg/m^2^	26.8 ± 5.8 (451)	29.5 ± 6.8 (85)	26.3 ± 4.0 (40)	.0003
Serum creatinine, mg/dL	1.8 ± 1.0 (449)	2.2 ± 2.5 (88)	1.4 ± 0.6 (40)	.003
Creatinine clearance, mL/min	47.3 ± 23.4 (446)	56.8 ± 40.0 (86)	56.5 ± 27.7 (40)	.003
Etiology of cardiomyopathy				
Ischemic	304 (66.5)	28 (31.8)	24 (60.0)	<.0001
Nonischemic	153 (33.5)	60 (68.2)	16 (40.0)	<.0001
NYHA class				.24
I	1 (0.0)	0 (0.0)	0 (0.0)	
II	174 (38.1)	31 (35.2)	20 (50.0)	
III	245 (53.7)	46 (52.3)	19 (47.5)	
IV	36 (7.9)	11 (12.5)	1 (2.5)	
HFH within 12 mo	258 (56.5)	56 (63.6)	23 (57.5)	.46
Prior CRT	177 (38.7)	25 (28.4)	14 (35.0)	.18
Prior ICD	125 (27.4)	41 (46.6)	16 (40.0)	.001
BNP, pg/mL	1066 ± 1230 (304)	855 ± 967 (66)	785 ± 578 (25)	.25
NT-proBNP, pg/mL	5835 ± 8083 (123)	5784 ± 7179 (19)	4125 ± 3754 (11)	.78
KCCQ score	51.8 ± 22.2 (455)	51.4 ± 24.7 (87)	57.6 ± 26.3 (40)	.29
Severity of mitral regurgitation				
Moderate-to-severe (grade 3+)	260 (56.9)	27/87 (31.0)	21 (52.5)	<.0001
Severe (grade 4+)	197 (43.1)	60/87 (69.0)	19 (47.5)	<.0001
EROA, cm^2^	0.41 ± 0.26 (439)	0.43 ± 0.25 (86)	0.39 ± 0.12 (40)	.31
Mitral valve area, cm^2^	5.2 ± 1.2 (437)	5.2 ± 1.3 (84)	4.7 ± 0.7 (38)	.06
Mitral valve gradient, mm Hg	2.3 ± 1.0 (326)	2.7 ±1.3 (64)	2.3 ± 1.0 (30)	.008
LVESD, cm	5.3 ± 0.8 (454)	5.5 ± 1.0 (87)	5.3 ± 1.0 (37)	.21
LVEDD, cm	6.2 ± 0.7 (454)	6.4 ± 0.8 (87)	6.2 ± 0.7 (38)	.06
LVESV, mL	133.0 ± 59.0 (430)	146.1 ± 60.7 (84)	141.4 ± 50.3 (34)	.15
LVEDV, mL	190.4 ± 71.4 (430)	208.3 ± 77.3 (84)	193.4 ± 60.2 (34)	.11
LVEF, %	31.6 ± 9.5 (430)	30.7 ± 8.7 (84)	27.6 ± 8.4 (34)	.046
RVSP, mm Hg	43.4 ± 12.9 (391)	45.7 ± 14.6 (79)	48.9 ± 18.6 (33)	.046

Data are presented as mean ± SD (No. patients), n (%), or n/N (%).

BNP, B-type natriuretic peptide; CABG, coronary artery bypass graft surgery; COPD, chronic obstructive pulmonary disease; CRT, cardiac resynchronization therapy (pacemaker or defibrillator); EROA, effective regurgitant orifice area (by PISA); HFH, heart failure hospitalization; ICD, implantable cardiac defibrillator; KCCQ, Kansas City Cardiomyopathy Questionnaire; LVEF, left ventricular ejection fraction; LVEDD, left ventricular end-diastolic dimension; LVESD, left ventricular end-systolic dimension; LVEDV, left ventricular end-diastolic volume; LVESV, left ventricular end-systolic volume; NT-pro-BNP, N-terminal B-type natriuretic peptide; NYHA, New York Heart Association; PCI, percutaneous coronary intervention; RVSP, right ventricular systolic pressure; STS, Society of Thoracic Surgeons; TIA, transient ischemic attack.

Baseline core-laboratory echocardiographic characteristics by race and ethnicity pooled across treatments and separately by treatment are presented in Table 1 and Supplemental Table S4, respectively. Black patients presented with a higher proportion of severe (4+) MR compared with White and Hispanic patients, although the mean EROA was not significantly different between groups. Mean mitral gradients were slightly higher in Black patients compared with other groups. Significant differences between groups were also observed for LVEF and right ventricular systolic pressure, whereas left ventricular volumes were similar.

### Safety outcomes

Supplemental Table S5 presents safety outcomes grouped by race and ethnicity. Adverse safety outcomes at 30 days occurred in 3 (1.4%) White patients, 1 (2.4%) Black patient, and 0 (0%) Hispanic patients treated with M-TEER (*P* = .62). Device-related complication rates were similar across race and ethnicity groups.

### Effectiveness outcomes

**Figure 1:**
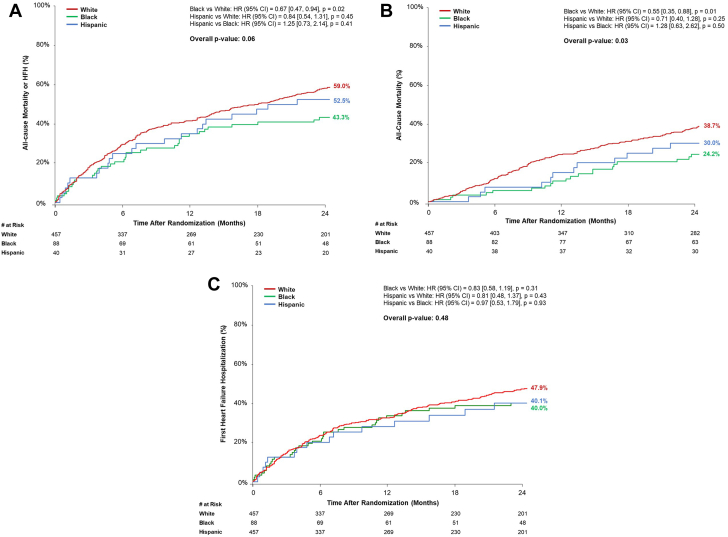
**Clinical outcomes at 2 years by race and ethnicity.** (**A**) The composite of all-cause mortality or HFH; (**B**) all-cause mortality; (**C**) time-to-first HFH. HFH, heart failure hospitalization.

Rates of the 2-year primary composite outcome and its components by race and ethnicity group are presented in Supplemental Table S6 and Figure 1A-C. By multivariable analysis in all patients, the 2-year risk of the composite of mortality or HFH was lower in Black patients compared with that in White patients (adjusted HR, 0.68; 95% CI, 0.47-0.98; *P* = .04) and similar in Hispanic patients compared to White patients (adjusted HR, 1.01; 95% CI, 0.63-1.64; *P* = .96) (Supplemental Table S7). Similar patterns for clinical outcomes by race and ethnicity were observed at 5-year follow-up (Supplemental Table S8).

**Figure 2:**
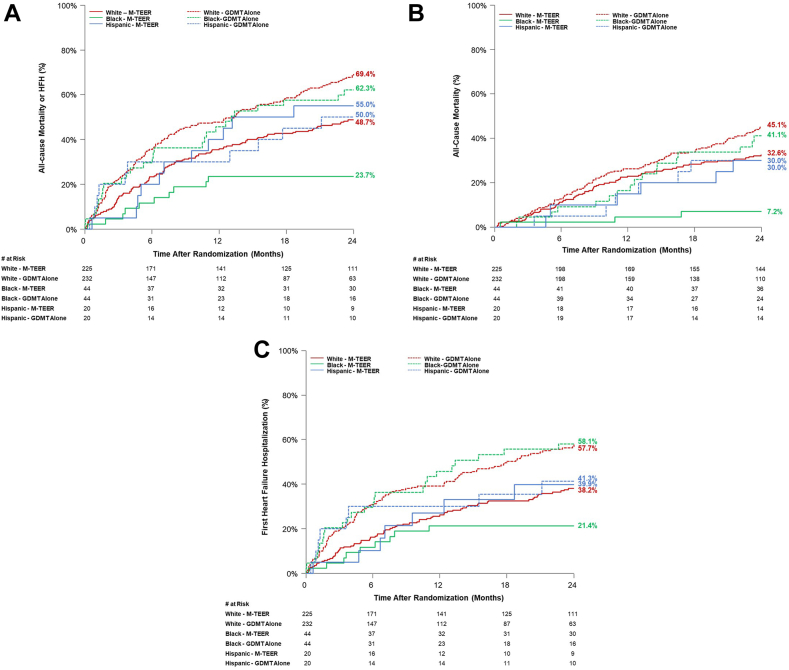
**Clinical outcomes at 2 years by race and ethnicity and treatment arm.** (**A**) The composite of all-cause mortality or HFH; (**B**) all-cause mortality; (**C**) time-to-first HFH. GDMT, guideline-directed medical therapy; HFH, heart failure hospitalization; M-TEER, mitral transcatheter edge-to-edge repair.

Two-year outcomes by race and ethnicity and treatment group are presented in Table 2, Figure 2A-C, and Central Illustration. Reductions in the primary composite outcome of all-cause mortality or HFH at 2 years were observed with MitraClip therapy plus GDMT compared with GDMT alone in White (48.7% vs 69.4%; HR, 0.59; 95% CI, 0.46-0.75) and Black (23.7% vs 62.3%; HR, 0.30; 95% CI, 0.14-0.61) patients, but not in Hispanic patients (50.0% vs 55.0%; HR, 1.09; 95% CI, 0.46-2.57; *P*_interaction_ = .06). A similar pattern was present for the reductions in the separate outcomes of all-cause mortality and first HFH (*P*_interaction_ = .05 and .16, respectively) (Table 2). Excluding Hispanic patients, no significant interaction between Black and White patients, randomization to MitraClip therapy, and the primary composite outcome was observed at 2 years (*P* = .06).

**Table 2:** Clinical outcomes at 2 years by race and ethnicity and treatment.

Outcome	White			Black			Hispanic			*P*_interaction_
	M-TEER (n = 225)	GDMT alone (n = 232)	HR (95% CI)	M-TEER (n = 44)	GDMT alone (n = 44)	HR (95% CI)	M-TEER (n = 20)	GDMT alone (n = 20)	HR (95% CI)	
All-cause mortality or HFH	108 (48.7)	154 (69.4)	0.59 (0.46-0.75)	10 (23.7)	27 (62.3)	0.30 (0.14-0.61)	11 (55)	10 (50)	1.09 (0.46-2.57)	.06
All-cause mortality	72 (32.6)	98 (45.1)	0.69 (0.51-0.94)	3 (7.2)	17 (41.1)	0.15 (0.04-0.51)	6 (30)	6 (30)	0.97 (0.31-3.00)	.05
Cardiovascular	55 (26.4)	78 (37.4)	0.66 (0.47-0.94)	3 (7.2)	16 (39.1)	0.16 (0.05-0.54)	4 (21.4)	5 (26.3)	0.78 (0.21-2.92)	.09
HF related	25 (13.2)	51 (26.2)	0.46 (0.29-0.74)	3 (7.2)	9 (24.6)	0.28 (0.08-1.04)	2 (12.2)	3 (16.1)	0.66 (0.11-3.93)	.71
Non-HF related	30 (15.2)	27 (15.2)	1.05 (0.62-1.76)	0 (0.0)	7 (19.3)	—	2 (10.5)	12.2 (2.0)	0.97 (0.14-6.89)	.91
Noncardiovascular	18 (9.0)	23 (13.8)	0.73 (0.4-1.36)	0 (0.0)	1 (3.2)	—	2 (10.9)	1 (5.0)	1.88 (0.17-20.8)	.74
All-cause Hospitalization	152 (70.1)	177 (82)	0.78 (0.63-0.97)	27 (64.5)	36 (83.4)	0.64 (0.39-1.06)	12 (60)	13 (66.3)	0.77 (0.35-0.68)	.78
HF related	77 (38.2)	118 (57.7)	0.55 (0.41-0.73)	9 (21.4)	25 (58.1)	0.29 (0.14-0.63)	8 (41.3)	7 (39.9)	0.85 (0.31-2.36)	.16

Rates are Kaplan-Meier estimates presented as n (%).

CI, confidence interval; HF, heart failure; HFH, heart failure hospitalization; HR, hazard ratio; *P*_intereaction_ = the *P* value for interaction between the race or ethnicity and randomization for each 2-y outcome.

**Central Illustration:**
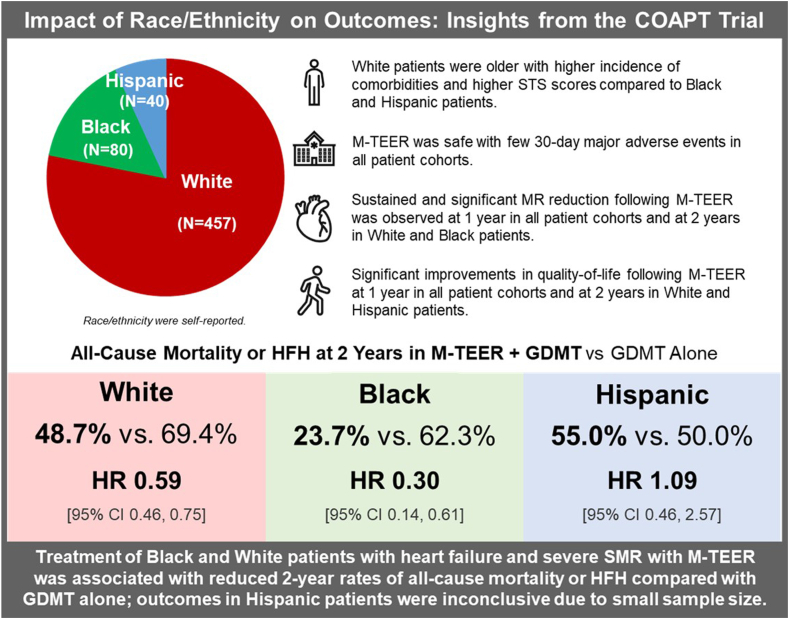
**Impact of race and ethnicity on outcomes from the COAPT trial.** Race and ethnicity were self-reported by patients in COAPT. Overall outcomes of the analysis are shown. Significant differences in baseline characteristics were noted between the groups, yet M-TEER was safe and effective in all cohorts. COAPT, Cardiovascular Outcomes Assessment of the MitraClip Percutaneous Therapy for Heart Failure Patients with Functional Mitral Regurgitation; GDMT, guideline-directed medical therapy; HFH, heart failure hospitalization; M-TEER, mitral transcatheter edge-to-edge repair; STS, Society of Thoracic Surgeons.

After multivariable adjustment, the 2-year risk of the composite of death or HFH was similar in Black and Hispanic patients compared with White patients treated with GDMT alone (Table 3). Conversely, after M-TEER, the risk of the 2-year primary composite outcome was lower in Black patients but higher in Hispanic patients compared with that of White patients (Table 3). Clinical outcomes by race and ethnicity group and treatment arm at 5 years are shown in Supplemental Table S9.

**Table 3:** Univariable and multivariable predictors of the 2-year risk of the composite of all-cause mortality or HF hospitalization by treatment

Variable	GDMT alone				M-TEER			
	Univariable		Multivariable		Univariable		Multivariable	
	HR (95% CI)	*P*	HR (95% CI)	*P*	HR (95% CI)	*P*	HR (95% CI)	*P*
Black (vs White)	0.87 (0.58-1.30)	.49	1.16 (0.73-1.83)	.53	0.42 (0.22-0.81)	.009	0.38 (0.19-0.76)	.007
Hispanic (vs White)	0.63 (0.33-1.19)	.15	0.67 (0.34-1.34)	.26	1.15 (0.62-2.13)	.67	2.05 (1.05-4.02)	.04
BNP or NT-proBNP (per 250 pg/mL BNP)^a^	1.06 (1.04-1.09)	<.0001	1.06 (1.03-1.08)	<.0001	1.07 (1.04-1.11)	<.0001	1.09 (1.04-1.13)	<.0001
6MWD (per 10.0 m)	0.98 (0.97-1.00)	.01	0.98 (0.97-1.00)	.02	0.98 (0.96-0.99)	.0009	—	—
Prior stroke	0.62 (0.38-1.00)	.05	0.54 (0.31-0.93)	.03	0.65 (0.36-1.17)	.15	—	—
Systolic blood pressure (per 10 mm Hg)	0.91 (0.83-1.00)	.045	0.87 (0.79-0.97)	.01	1.01 (0.91-1.12)	.90	—	—
Baseline use of vasodilators	1.50 (1.08-2.09)	.02	—	—	1.94 (1.31-2.88)	.001	2.44 (1.59-3.76)	<.0001
Prior atrial fibrillation	1.20 (0.91-1.59)	.19	—	—	1.57 (1.10-2.23)	.01	1.89 (1.27-2.81)	.002
KCCQ score (per 5 points)	0.97 (0.94-1.00)	.06	—	—	0.95 (0.91-0.98)	.005	0.94 (0.90-0.98)	.0005
Peripheral vascular disease	0.89 (0.61-1.28)	.52	—	—	1.96 (1.32-2.90)	.0008	2.08 (1.35-3.19)	.0008
Body mass index (per 1 kg/m^2^)	0.99 (0.97-1.02)	.52	—	—	1.03 (1.00-1.06)	.055	1.04 (1.01-1.07)	.008

BNP, B-type natriuretic peptide; CI, confidence interval; GDMT, guideline-directed medical therapy; HF, heart failure; HR, hazard ratio; M-TEER, mitral-transcatheter edge-to-edge repair; NT-proBNP, N-terminal prohormone B-type natriuretic peptide; 6MWD, 6-minute walk distance; KCCQ, Kansas City Cardiomyopathy Questionnaire.

a If only baseline NT-proBNP was measured, NT-proBNP was converted to BNP by dividing by 7. Race and ethnicity were forced into the model.

### Changes in MR and QoL

**Figure 3:**
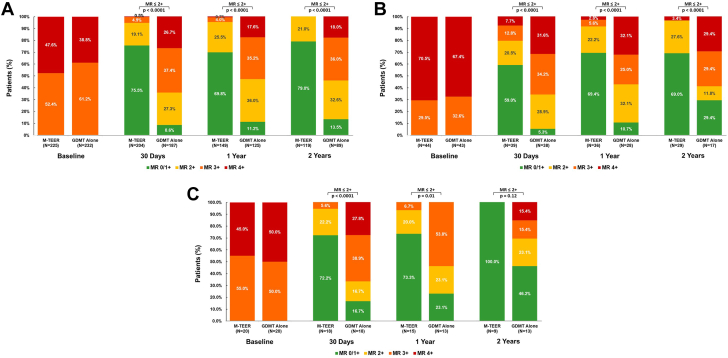
**Severity of mitral regurgitation during 2-year follow-up by race and ethnicity and treatment arm**. (**A**) White patients; (**B**) Black patients; (**C**) Hispanic patients. GDMT, guideline-directed medical therapy; MR, mitral regurgitation; M-TEER, mitral transcatheter edge-to-edge repair.

At 2 years, MR grade ≤2+ was present more often after M-TEER compared with GDMT alone in White (119/119 [100.0%] vs 41/89 [46.1%]; *P* < .001) and Black (28/29 [96.6%] vs 7/17 [41.2%]; *P* < .001) patients but not in Hispanic patients (9/9 [100.0%] vs 9/13 [69.2%]; *P* = .12) (Figure 3A-C). However, among Hispanic patients MR grade ≤2+ was more common in the M-TEER group at 1 year (14/15 [93.3%] vs 6/13 [46.2%]; *P* = .01).

**Figure 4:**
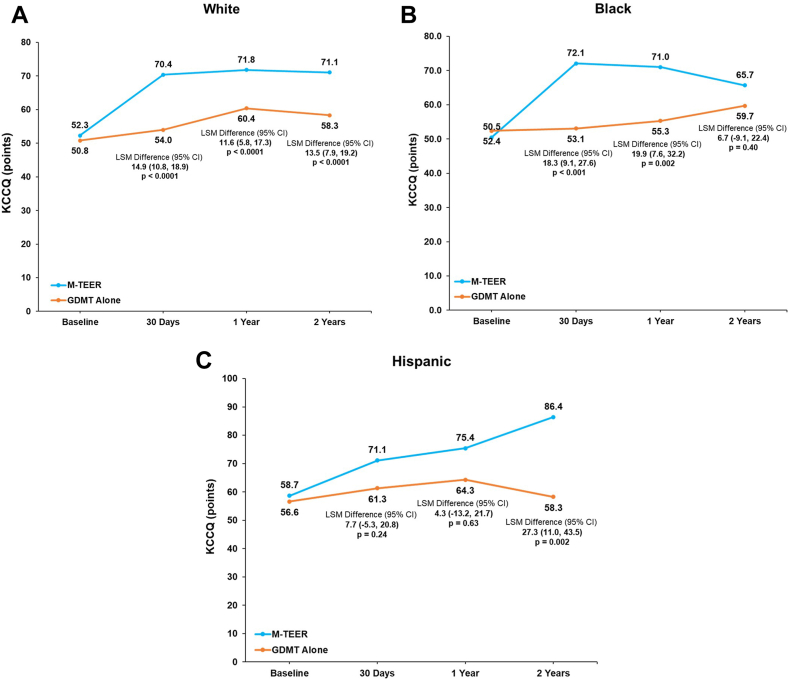
**KCCQ score during 2-year follow-up by race and ethnicity and treatment arm.** (**A**) White patients; (**B**) Black patients; (**C**) Hispanic patients. GDMT, guideline-directed medical therapy; KCCQ, Kansas City Cardiomyopathy Questionnaire; LSM, least square means; M-TEER, mitral transcatheter-edge-to-edge repair.

The mean change in KCCQ score from baseline to 2 years in patients randomized to M-TEER versus GDMT alone was 17.3 ± 25.6 vs 2.7 ± 24.5, respectively, in White patients (least squares mean [LSM] difference, 13.5; 95% CI, 7.9-19.2; *P* < .0001); 15.2 ± 27.9 vs 7.4 ± 34.8, respectively, in Black patients (LSM difference, 6.7; 95% CI, −9.1 to 22.4; *P* = .40); and 27.1 ± 22.5 vs 0.3 ± 20.8, respectively, in Hispanic patients (LSM difference, 27.3; 95% CI, 11.0-43.5; *P* = .002) (Figure 4A-C). However, among Black patients, QoL was superior after M-TEER at 1 year (LSM difference, 19.9; 95% CI, 7.6-32.2; *P* = .002).

## Discussion

This analysis of 585 patients from the COAPT trial represents the largest study to date evaluating outcomes after M-TEER in patients with HF and with severe SMR by race and ethnicity using high-quality data from a randomized clinical trial. The principal findings of this study are as follows: (1) baseline clinical characteristics and surgical risk varied substantially by race and ethnicity among patients enrolled in COAPT; (2) MitraClip treatment was safe, with few complications noted within 30 days in all patients; (3) freedom from all-cause mortality or HFH through 2-year follow-up was improved with MitraClip treatment compared with GDMT alone in White patients and to an even greater extent in Black patients, but not in Hispanic patients, although the small sample size of this group precludes a definitive conclusion; (4) MitraClip treatment led to sustained reductions in MR grade through 2 years in White and Black patients; the reduction in MR reduction in the smaller group of Hispanic patients was significant at 1 year but not at 2 years; (5) MitraClip treatment also improved QoL as measured by the change in KCCQ score from baseline to 2 years in White and Hispanic but not in Black patients, although in Black patients, QoL was improved with M-TEER through 1 year follow-up.

Racial and ethnic disparities in access to cardiovascular treatments and outcomes have been previously described.^1^ An analysis from the STS/Transcatheter Valve Therapy Registry demonstrated that minority patients accounted for <10% of patients undergoing transcatheter aortic valve replacement in the United States, suggesting significant underrepresentation of such patients.^4^ Similarly, an analysis of the National Inpatient Sample demonstrated that utilization rates across all structural heart procedures (including transcatheter aortic valve replacement, transcatheter mitral valve repair, and left atrial appendage occlusion) were significantly lower in Black and Hispanic patients compared with those in White patients.^3^ Studies have also suggested that minority group patients are less likely to undergo transcatheter mitral valve therapies. A recent analysis of 1567 patients demonstrated that Black and Hispanic patients were 59% (adjusted odds ratio [OR], 0.41; *P* < .001) and 51% (adjusted OR, 0.49; *P* < .001), respectively, less likely to undergo M-TEER procedures compared with White patients.^11^ Other studies have consistently demonstrated disparities in access to M-TEER and transcatheter mitral valve replacement in minority patients.^12^ In most of these studies, <10% of treated patients were Black or Hispanic, a proportion that has not significantly changed over time.^7^ Barriers to care and access to transcatheter therapies for minority patients include delayed diagnosis of valvular heart disease, referral bias, lack of trust in the health care system due to historical issues in care delivery, socioeconomic disparities, geographic proximity, and transportation. These considerations have resulted in a lack of diversity in clinical trials limiting the generalization of intervention effectiveness to minority patients.^13^, ^14^, ^15^ Given the public health implications of the lack of minority group representation in clinical trials, the Food and Drug Administration has introduced new guidance to enhance enrollment of participants from underrepresented populations in future studies.^16^

Beyond issues of access, little is known regarding the outcomes of minority group patients after mitral valve therapies. A recent analysis of 458 patients from the VA Healthcare System showed similar outcomes among White, Black, and Hispanic patients, although a small sample size precluded any definitive conclusions in the Hispanic group.^17^ In the randomized COAPT trial, the proportion of traditionally underrepresented patients (22%) was higher than in previous analyses in the literature. Strengths of the present analysis include detailed assessments of baseline clinical variables, core laboratory assessed echocardiographic data, and adjudicated long-term clinical events stratified by race and ethnicity, not previously available from prior studies. Baseline characteristics substantially varied between the groups, with White patients being older and having the highest STS risk scores for predicted mortality after surgical mitral valve repair and replacement. Conversely, Black patients were more likely to be female and have nonischemic cardiomyopathy, while Hispanic patients were the least symptomatic.

M-TEER with the MitraClip device had variable effectiveness across the 3 groups. Specifically, the 2-year rates of death and HFH (before crossovers being allowed in GDMT alone group patients) were lower with M-TEER in White and Black patients, but not in Hispanic patients. Notably, when excluding Hispanic patients, the interaction analysis between Black and White patients, randomization to MitraClip therapy, and 2-year rates of the primary composite outcome did not meet statistical significance. Similarly, the presence of severe MR reduced significantly at 2 years by M-TEER compared with GDMT alone in White and Black but not in Hispanic patients. However, MR reduced significantly by M-TEER in Hispanic patients at 30 days and 1 year, and 100% of surviving Hispanic patients treated with M-TEER had MR ≤2+ at 2 years. The lack of significance in clinical effectiveness and MR reduction at 2 years after M-TEER in Hispanic patients is potentially due to the small sample size of this group; further studies are required to confirm or refute these observations. Perhaps more notably, the efficacy of M-TEER in reducing death and HFH was greater in Black patients than White (or Hispanic) patients; as a result, Black patients treated with M-TEER had the lowest 2-year risk of death or HFH. While Black patients had fewer comorbidities, lower rates of the primary end point were observed even after adjustment for confounding baseline clinical and echocardiographic characteristics. Moreover, as abovementioned, Black patients were also more likely to have nonischemic cardiomyopathy, and whether this contributed to observed rates of outcomes in this analysis must be considered. While a meta-analysis of Chiarito et al^18^ suggested lower rates of mortality after M-TEER in patients with nonischemic (vs ischemic) cardiomyopathy, the findings of COAPT suggested no significant difference in the primary end point at 5 years when stratified by the etiology of cardiomyopathy.^8^^,^^18^ Therefore, it seems less likely the greater efficacy of M-TEER in Black patients was a result of the prevalence of nonischemic cardiomyopathy in this population. Additionally, whether baseline GDMT regimens may have impacted the benefit observed with M-TEER in Black patients in this study merits further evaluation. Thus, the mechanism(s) for a potentially greater benefit in Black patients are not immediately clear, and these findings should also be examined in future studies. Nonetheless, it is reassuring that the outcomes of M-TEER in Black patients appear to be at least as good as in White patients, in contrast to what has been described with percutaneous coronary intervention.^19^ Finally, QoL as measured by improvement in KCCQ score after M-TEER compared with GDMT alone was greatest at 2 years in White and Hispanic patients. However, at 1 year, Black patients had the greatest improvement in QoL after M-TEER, an effect that was diminished with longer-term follow-up. Again, whether there is a true loss in symptomatic improvement after M-TEER in Black patients with long-term follow-up deserves further study. In summary, these data demonstrate that in the COAPT trial, traditionally underrepresented groups with symptomatic HF and severe SMR derived substantial benefits from M-TEER therapy.

### Limitations

First, the present study is a post hoc analysis from a prospective randomized trial. The number of patients enrolled who were Black and (particularly) Hispanic was modest, and randomization was not stratified by race or ethnicity. Thus, although the baseline features appeared balanced within the randomized groups and outcomes were adjusted for numerous prognostic baseline clinical and echocardiographic characteristics, residual confounding cannot be excluded. These results should thus be considered hypothesis generating. Second, race and ethnicity were patient reported and prospectively collected as a single mutually exclusive variable in the electronic case report form. Therefore, we were limited in our ability to analyze patients by race or ethnicity as separate categories. Third, although KCCQ score as a measure of health-related QoL has been rigorously validated and independently associated with prognosis in patients with HF,^20^ perceived health-related QoL captured by the KCCQ score may differ by race and ethnicity.^21^ For example, in the present study, Hispanic patients were the least symptomatic at baseline by the KCCQ score without obvious explanation. The results of racial and ethnic differences in QoL as reflected by KCCQ scores reported in this study should be interpreted in the context of this limitation. Fourth, the most reliable assessment of M-TEER’s effects in the COAPT trial were within the first 2 years, before the GDMT alone group was permitted to receive M-TEER treatment. While 5-year outcomes are included as a sensitivity analysis, the effectiveness of M-TEER in the COAPT trial diminished after the 2-year mark due to this factor.^8^ Finally, the race and ethnicity of the COAPT trial were broadly representative of the US population. However, non-Black and non-Hispanic races and ethnic subgroups (eg, Asian patients) were not included in this analysis given their low enrollment, and therefore, results from this study may not be generalizable to these other groups.

## Conclusions

Severe SMR was safely reduced in Black, White, and Hispanic patients enrolled in the COAPT trial. All 3 groups also experienced lower rates of the 2-year primary composite outcome of all-cause mortality or HFH, with the greatest benefits noted in Black patients. Future studies in larger patient datasets are needed to corroborate these findings in traditionally underrepresented patients with SMR.
