# Supplementary material for: Impact of Race and Ethnicity on Outcomes After Mitral Transcatheter Edge-to-Edge Repair: Analysis From the COAPT Trial
Source: J Soc Cardiovasc Angiogr Interv. 2025 Oct 30;5(3 Suppl):103822. doi: 10.1016/j.jscai.2025.103822 (PMC13112807; doi:10.1016/j.jscai.2025.103822)
Supplement: Supplemental Tables 1-9 [file mmc1.docx]

**SUPPLEMENTARY DATA**

**Impact of Race and Ethnicity on Outcomes After Transcatheter Edge-to-Edge Repair:
Analysis from the COAPT Trial**

|  | **Page** |
| --- | --- |
| **Supplementary Table 1.** Distribution of all Race and Ethnicity Categories in COAPT Trial and their Respective Prevalence in the United States | 2 |
| **Supplementary Table 2.** Baseline Clinical Characteristics by Race and Ethnicity and Treatment Arm | 3 |
| **Supplementary Table 3.** Baseline Medication Usage by Race and Ethnicity | 5 |
| **Supplementary** **Table 4.** Baseline Echocardiographic Variables by Race and Ethnicity and Treatment Arm | 7 |
| **Supplementary Table 5.** Safety Endpoints at 30 days in the M-TEER Group by Race and Ethnicity | 8 |
| **Supplementary Table 6.** Clinical Outcomes at Two Years by Race and Ethnicity | 9 |
| **Supplementary Table 7.** Multivariable Predictors of the Two-year Risk of the Composite of All-cause Mortality or Heart Failure Hospitalization | 10 |
| **Supplementary Table 8.** Clinical Outcomes at Five Years by Race and Ethnicity | 11 |
| **Supplementary Table 9.** Clinical Outcomes at Five Years by Race and Ethnicity and Treatment | 12 |

**Supplementary Table 1.** Distribution of all Race and Ethnicity Categories in COAPT Trial and their Respective Prevalence in the United States

|  | **Patients Enrolled in COAPT  (N=614)** | **Representation in U.S. Population (N=331,449,281)*** |
| --- | --- | --- |
| White or Caucasian | 457 (74.4) | 204,277,273 (61.6) |
| Black or African American | 88 (14.3) | 41,104,200 (12.4) |
| Hispanic or Latino | 40 (6.5) | 62,080,044 (18.7) |
| Asian | 18 (2.9) | 19,886,049 (6.0) |
| Native Hawaiian or Other Pacific Islander | 1 (0.2) | 689,966 (0.2) |
| American Indian or Alaska Native | 0 (0.0) | 3,727,135 (1.1) |
| Other | 10 (1.6) | N/A |

*Based on 2020 decennial United States census from census.gov. Data are presented as n (%).

**Supplementary Table 2.** Baseline Clinical Characteristics by Race and Ethnicity and Treatment Arm

|  | **White** | | **Black** | | | **Hispanic** | | | |  |
| --- | --- | --- | --- | --- | --- | --- | --- | --- | --- | --- |
|  | **M-TEER (N=225)** | **GDMT Alone  (N=232)** | | **M-TEER (N=44)** | **GDMT Alone (N=44)** | | **M-TEER (N=20)** | **GDMT Alone (N=20)** | | |
| Age, years | 73.6 ± 10.4 | 74.1 ± 6.4 | | 63.6 ± 12.6 | 67.2 ± 11.8 | | 66.1 ± 14.4 | 70.8 ± 15.6 | | |
| Male sex | 157 (69.8) | 146 (62.9) | | 19 (43.2) | 20 (45.5) | | 15 (75.0) | 14 (70.0) | | |
| Diabetes mellitus | 71 (31.6) | 84 (36.2) | | 20 (45.5) | 21 (47.7) | | 8 (40.0) | 11 (55.0) | | |
| Hypertension | 180 (80.0) | 187 (80.6) | | 37 (84.1) | 38 (86.4) | | 16 (80.0) | 15 (75.0) | | |
| Hypercholesterolemia | 133 (59.1) | 126 (54.3) | | 17 (38.6) | 21 (47.7) | | 8 (40.0) | 9 (45.0) | | |
| Peripheral vascular disease | 46 (20.4) | 50 (21.6) | | 6 (13.6) | 5 (11.4) | | 0 (0.0) | 1 (5.0) | | |
| History of anemia | 47 (20.9) | 51 (22.0) | | 13 (29.5) | 15 (34.1) | | 4 (20.0) | 8 (40.0) | | |
| History of atrial fibrillation/flutter | 141 (62.7) | 128 (55.2) | | 18 (40.9) | 22 (50.0) | | 6 (30.0) | 7 (35.0) | | |
| Coronary artery disease | 174 (77.3) | 180 (77.6) | | 21 (47.7) | 24 (54.5) | | 13 (65.0) | 15 (75.0) | | |
| Prior myocardial infarction | 121 (53.8) | 131 (56.5) | | 15 (34.1) | 17 (38.6) | | 11 (55.0) | 6 (30.0) | | |
| Prior PCI | 106 (47.1) | 124 (53.4) | | 10 (22.7) | 13 (29.5) | | 6 (30.0) | 9 (45.0) | | |
| Prior CABG | 102 (45.3) | 107 (46.1) | | 7 (15.9) | 8 (18.2) | | 7 (35.0) | 7 (35.0) | | |
| Prior stroke or TIA | 38 (16.9) | 32 (13.8) | | 14 (31.8) | 9 (20.5) | | 2 (10.0) | 6 (30.0) | | |
| COPD | 58 (25.8) | 53 (22.8) | | 12 (27.3) | 13 (29.5) | | 0 (0.0) | 2 (10.0) | | |
| STS risk score for replacement | 8.5 ± 5.6 | 8.9 ± 6.4 | | 5.5 ± 4.1 | 7.0 ± 4.5 | | 5.9 ± 5.7 | 7.9 ± 5.9 | | |
| ≥ 8% | 107 (47.6) | 103 (44.4) | | 11 (25.0) | 18 (40.9) | | 3 (15.0) | 10 (50.0) | | |
| STS risk score for repair | 6.0 ± 4.9 | 6.4 ± 5.7 | | 4.6 ± 8.9 | 4.4 ± 3.2 | | 3.6 ± 4.1 | 5.2 ± 4.6 | | |
| ≥ 8% | 57 (25.3) | 58 (25.0) | | 4 (9.1) | 7 (15.9) | | 2 (10.0) | 3 (15.0) | | |
| Body mass index, kg/m^2^ | 26.8 ± 5.9 (223) | 26.7 ± 5.7 (228) | | 28.7 ± 6.5 (44) | 30.5 ± 7.2 (41) | | 26.7 ± 3.2 (20) | 26.0 ± 4.6 (20) | | |
| Creatinine, mg/dL | 1.8 ± 1.2 (223) | 1.7 ± 0.9 (226) | | 1.9 ± 1.8 (44) | 2.5 ± 3.0 (44) | | 1.3 ± 0.5 | 1.5 ± 0.7 | | |
| Creatinine clearance, mL/min | 47.4 ± 23.8 (222) | 47.2 ± 23.1 (224) | | 64.6 ± 42.8 (44) | 48.7 ± 35.4 (42) | | 61.5 ± 30.0 (20) | 51.5 ± 25.0 (20) | | |
| Etiology of cardiomyopathy | | | | | | | | |  |  |
| Ischemic | 151 (67.1) | 153 (65.9) | | 14 (31.8) | 14 (31.8) | | 10 (50.0) | 14 (70.0) | | |
| Non-ischemic | 74 (32.9) | 79 (34.1) | | 30 (68.2) | 30 (68.2) | | 10 (50.0) | 6 (30.0) | | |
| NYHA class | | | | | | | | |  |  |
| I | 1 (0.4) | 0 (0.0) | | 0 (0.0) | 0 (0.0) | | 0 (0.0) | 0 (0.0) | | |
| II | 99 (44.0) | 75 (32.5) | | 11 (25) | 20 (45.5) | | 11 (55.0) | 9 (45.0) | | |
| III | 114 (50.7) | 131 (56.7) | | 27 (61.4) | 19 (43.2) | | 8 (40.0) | 11 (55.0) | | |
| IV | 11 (4.9) | 25 (10.8) | | 6 (13.6) | 5 (11.4) | | 1 (5.0) | 0 (0.0) | | |
| HFH within 12 months | 127 (56.4) | 131 (56.5) | | 29 (65.9) | 27 (61.4) | | 12 (60.0) | 11 (55.0) | | |
| Prior CRT | 91 (40.4) | 86 (37.1) | | 11 (25.0) | 14 (31.8) | | 8 (40.0) | 6 (30.0) | | |
| Prior ICD | 56 (24.9) | 69 (29.7) | | 23 (52.3) | 18 (40.9) | | 8 (40.0) | 8 (40.0) | | |
| BNP, pg/mL | 1040.5 ± 1108.1 (149) | 1089.7 ± 1339.5 (155) | | 938.6 ± 1253.0 (34) | 767.1 ± 524.8 (32) | | 887.8 ± 534.4 (13) | 674.3 ± 625.4 (12) | | |
| NT-proBNP, pg/mL | 5631.5 ± 7051.1 (61) | 6036.1 ± 9037.6 (62) | | 3772.4 ± 3358.7 (7) | 6957.2 ± 8608.4 (12) | | 2485.0 ± 1802.2 (5) | 5490.7 ± 4545.5 (6) | | |
| KCCQ score | 52.8 ± 21.9 (225) | 50.8 ± 22.6 (230) | | 50.5 ± 24.8 (44) | 52.4 ± 24.9 (43) | | 58.7 ± 26.4 (20) | 56.6 ± 26.8 (20) | | |

Data presented as mean ± SD (number of measures) or n (%). BNP = B-type natriuretic peptide; CABG = coronary artery bypass graft surgery; COPD = chronic obstructive pulmonary disease; CRT = cardiac resynchronization therapy (pacemaker or defibrillator); HFH = heart failure hospitalization; ICD = implantable cardiac defibrillator; KCCQ = Kansas City Cardiomyopathy Questionnaire; NT-pro-BNP = N-terminal B-type natriuretic peptide; NYHA = New York Heart Association; PCI = percutaneous coronary intervention; STS = Society of Thoracic Surgeons; TIA = transient ischemic attack.

**Supplementary Table 3.** Baseline Medication Usage by Race and Ethnicity

|  | **White  (N=457)** | **Black (N=88)** | **Hispanic  (N=40)** |
| --- | --- | --- | --- |
| Beta-blocker | 89.9% (411/457) | 89.8% (79/88) | 92.5% (37/40) |
| ACEI, ARB or ARNI | 66.3% (303/457) | 67.0% (59/88) | 82.5% (33/40) |
| ACEI | 41.6% (190/457) | 33.0% (29/88) | 62.5% (25/40) |
| ARB | 21.9% (100/457) | 30.7% (27/88) | 12.5% (5/40) |
| ARNI | 2.8% (13/457) | 3.4% (3/88) | 10.0% (4/40) |
| Mineralocorticoid receptor antagonist | 49.9% (228/457) | 45.5% (40/88) | 60.0% (24/40) |
| Nitrate | 5.9% (27/457) | 13.6% (12/88) | 7.5% (3/40) |
| Hydralazine | 14.4% (66/457) | 30.7% (27/88) | 12.5% (5/40) |
| Nitrate plus hydralazine | 4.6% (21/457) | 9.1% (8/88) | 7.5% (3/40) |
| Diuretic | 89.7% (410/457) | 86.4% (76/88) | 90.0% (36/40) |
| Chronic oral anticoagulant, any | 45.3% (207/457) | 38.6% (34/88) | 32.5% (13/40) |
| Warfarin | 30.4% (139/457) | 27.3% (24/88) | 25.0% (10/40) |
| Direct acting oral anticoagulant | 15.1% (69/457) | 11.4% (10/88) | 7.5% (3/40) |
| Aspirin | 61.1% (279/457) | 62.5% (55/88) | 70.0% (28/40) |
| P2Y12 receptor inhibitor, any | 25.4% (116/457) | 15.9% (14/88) | 25.0% (10/40) |
| Clopidogrel | 22.1% (101/457) | 13.6% (12/88) | 22.5% (9/40) |
| Prasugrel | 1.8% (8/457) | 1.1% (1/88) | 2.5% (1/40) |
| Ticagrelor | 1.5% (7/457) | 1.1% (1/88) | 2.5% (1/40) |
| Prasugrel or Ticagrelor | 3.3% (15/457) | 2.3% (2/88) | 5.0% (2/40) |
| Statin | 63.7% (291/457) | 51.1% (45/88) | 62.5% (25/40) |

Medications listed were based on usage at the date of baseline visit or at least one day during 30 days prior to reference start date

**Supplementary** **Table 4.** Baseline Echocardiographic Variables by Race and Ethnicity and Treatment Arm

|  | **White** | | **Black** | | **Hispanic** | |
| --- | --- | --- | --- | --- | --- | --- |
|  | **M-TEER  (N=225)** | **GDMT Alone  (N=232)** | **M-TEER (N=44)** | **GDMT Alone**  **(N=44)** | **M-TEER (N=20)** | **GDMT Alone**  **(N=20)** |
| Severity of mitral regurgitation | | | | | | |
| Moderate-to-severe, grade 3+ | 118 (52.4) | 142 (61.2) | 13 (29.5) | 14 (32.6) | 11 (55.0) | 10 (50.0) |
| Severe, grade 4+ | 107 (47.6) | 90 (38.8) | 31 (70.5) | 29 (67.4) | 9 (45.0) | 10 (50.0) |
| EROA, cm^2^ | 0.4 ± 0.2 (213) | 0.4 ± 0.2 (226) | 0.4 ± 0.2 (43) | 0.4 ± 0.1 (43) | 0.4 ± 0.1 (20) | 0.4 ± 0.1 (20) |
| Mitral valve area, cm^2^ | 5.2 ± 1.3 (214) | 5.2 ± 1.2 (223) | 5.2 ± 1.4 (41) | 5.1 ± 1.3 (43) | 4.6 ± 0.4 (18) | 4.8 ± 0.9 (20) |
| Mean mitral valve gradient, mmHg | 2.4 ± 1.0 (154) | 2.3 ± 1.0 (172) | 3.0 ± 1.3 (31) | 2.5 ± 1.2 (33) | 2.3 ± 1.2 (14) | 2.3 ± 0.8 (16) |
| LVESD, cm | 5.3 ± 0.9 (224) | 5.3 ± 0.8 (230) | 5.4 ± 0.9 (44) | 5.5 ± 1.0 (43) | 5.3 ± 0.7 (20) | 5.3 ± 1.2 (17) |
| LVEDD, cm | 6.2 ± 0.7 (224) | 6.2 ± 0.7 (230) | 6.3 ± 0.9 (44) | 6.4 ± 0.8 (43) | 6.1 ± 0.6 (20) | 6.2 ± 0.9 (18) |
| LVESV, mL | 134.0 ± 56.7 (207) | 132.0 ± 61.1 (223) | 147.3 ± 60.6 (43) | 144.8 ± 61.6 (41) | 142.6 ± 48.1 (18) | 140.1 ± 54.2 (16) |
| LVEDV, mL | 192.9 ± 68.4 (207) | 188.0 ± 74.2 (223) | 210.6 ± 80.9 (43) | 205.9 ± 74.3 (41) | 190.9 ± 61.2 (18) | 196.3 ± 61.1 (16) |
| LVEF, % | 31.8 ± 9.4 (207) | 31.5 ± 9.6 (223) | 30.4 ± 8.0 (43) | 31.0 ± 9.5 (41) | 25.6 ± 5.0 (18) | 29.8 ± 10.8 (16) |
| RVSP, mmHg | 43.1 ± 13.1 (188) | 43.7 ± 12.8 (203) | 44.1 ± 13.0 (39) | 47.3 ± 16.0 (40) | 49.3 ± 17.4 (16) | 48.5 ± 20.2 (17) |

Data are presented as mean ± SD (number of measures), n (%), or n/N(%). EROA = effective regurgitant orifice area (by PISA); GDMT = guideline-directed medical therapy; LVEF = left ventricular ejection fraction; LVEDD = left ventricular end-diastolic dimension; LVESD = left ventricular end-systolic dimension; LVEDV = left ventricular end-diastolic volume; LVESV = left ventricular end-systolic volume; M-TEER = mitral transcatheter edge-to-edge repair; RVSP = right ventricular systolic pressure.

**Supplementary Table 5.** Safety Endpoints at 30 Days in the M-TEER Group by Race and Ethnicity

|  | **White**  **(N=225)** | **Black**  **(N=44)** | **Hispanic**  **(N=20)** | **P-value** |
| --- | --- | --- | --- | --- |
| **Primary safety outcome*** | 3 (1.4) | 1 (2.4) | 0 (0.0) | 0.62 |
| **Device-related complications** | 3 (1.4) | 1 (2.4) | 0 (0.0) | 0.62 |
| Single leaflet device attachment | 1 (0.5) | 1 (2.4) | 0 (0.0) | 0.38 |
| Device embolization | 1 (0.5) | 0 (0.0) | 0 (0.0) | 0.99 |
| Endocarditis requiring surgery | 0 (0.0) | 0 (0.0) | 0 (0.0) | - |
| Mitral stenosis requiring surgery | 0 (0.0) | 0 (0.0) | 0 (0.0) | - |
| Any device-related complication requiring non-elective CV surgery | 1 (0.5) | 0 (0.0) | 0 (0.0) | 0.99 |
| **Progressive heart failure** | 0 (0.0) | 0 (0.0) | 0 (0.0) | - |
| Left ventricular assist device implant | 0 (0.0) | 0 (0.0) | 0 (0.0) | - |
| Heart transplant | 0 (0.0) | 0 (0.0) | 0 (0.0) | - |

Data are presented as n (%). *The composite of device-related complications or progressive heart failure events at 30 days. CV = cardiovascular.

**Supplementary Table 6.** Clinical Outcomes at Two Years by Race and Ethnicity

|  | **White**  **(n=457)** | **Black**  **(n=88)** | **Hispanic**  **(n=40)** | **Black vs.**  **White**  **HR [95% CI]** | **Hispanic vs. White**  **HR [95% CI]** | **Hispanic vs. Black**  **HR [95% CI]** |
| --- | --- | --- | --- | --- | --- | --- |
| All-cause mortality or HFH | 262 (59.0) | 37 (43.3) | 21 (52.5) | 0.67 [0.47, 0.94] | 0.84 [0.54, 1.31] | 1.25 [0.73, 2.14] |
| All-cause mortality | 170 (38.7) | 20 (24.2) | 12 (30.0) | 0.55 [0.35, 0.88] | 0.71 [0.4, 1.28] | 1.28 0.63, 2.62] |
| Cardiovascular | 129 (31.1) | 19 (23.3) | 9 (23.8) | 0.70 [0.43, 1.13] | 0.70 [0.36, 1.38] | 1.01 [0.46, 2.23] |
| Heart failure-related | 76 (19.7) | 12 (15.5) | 5 (14.2) | 0.75 [0.41, 1.37] | 0.66 [0.27, 1.63] | 0.88 [0.31, 2.51] |
| Non-heart failure-related | 94 (23.7) | 8 (10.5) | 7 (18.4) | 0.40 [0.2, 0.83] | 0.75 [0.35, 1.62] | 1.87 [0.68, 5.16] |
| Non-cardiovascular | 41 (11.1) | 1 (1.4) | 3 (8.2) | 0.12 [0.02, 0.84] | 0.74 [0.23, 2.38] | 0.33 [0.66, 60.81] |
| Hospitalization for any cause | 329 (76.0) | 63 (74.2) | 25 (62.8) | 0.94 [0.72, 1.23] | 0.78 [0.52, 1.17] | 0.83 [0.52, 1.32] |
| Heart failure-related | 195 (47.9) | 34 (40.0) | 15 (40.1) | 0.83 [0.58, 1.19] | 0.81 [0.48, 1.37] | 0.97 [0.53, 1.79] |

Rates are time-to-first event Kaplan-Meier estimates presented as n (%). Hazard ratios and 95% CIs are unadjusted values.

**Supplementary Table 7.** Multivariable Predictors of the Two-year Risk of the Composite of All-cause Mortality or Heart Failure Hospitalization

|  | **HR [95% CI]** | **P value** |
| --- | --- | --- |
| Black (vs. White) | 0.68 [0.47, 0.98] | 0.04 |
| Hispanic (vs. White) | 1.01 [0.63, 1.64] | 0.96 |
| BNP/NT-proBNP (per 250 pg/mL BNP)* | 1.06 [1.04, 1.09] | <0.0001 |
| Baseline use of vasodilators | 1.99 [1.50, 2.65] | <0.0001 |
| Prior atrial fibrillation | 1.53 [1.19, 1.96] | 0.0008 |
| EROA (per 10 mm^2^) | 1.10 [1.03, 1.18] | 0.005 |
| 6MWD (per 10 meters) | 0.99 [0.98, 1.00] | 0.01 |
| Prior stroke | 0.66 [0.44, 0.99] | 0.04 |
| Randomization to treatment with MitraClip | 0.51 [0.40, 0.65] | <0.0001 |

*If only baseline NT-proBNP was measured, NT-proBNP was converted to BNP by dividing by 7. Race and ethnicity were forced into the model. BNP = b-type natriuretic peptide, NT/proBNP = N-terminal pro-B-type natriuretic peptide, EROA = Effective regurgitant orifice area, 6MWD = 6-minute walking distance.

**Supplementary Table 8.** Clinical Outcomes at Five Years by Race and Ethnicity

|  | **White**  **(n=457)** | **Black**  **(n=88)** | **Hispanic**  **(n=40)** | **Black vs. White**  **HR [95% CI]** | **Hispanic vs. White**  **HR [95% CI]** | **Hispanic vs. Black**  **HR [95% CI]** |
| --- | --- | --- | --- | --- | --- | --- |
| All-cause mortality or HFH | 362 (83.4) | 62 (76.6) | 32 (81.0) | 0.77 [0.59, 1.00] | 0.87 [0.61, 1.25] | 1.11 [0.73, 1.71] |
| All-cause mortality | 276 (65.2) | 41 (53.9) | 22 (56.1) | 0.68 [0.49, 0.95] | 0.74 [0.48, 1.14] | 1.07 [0.63, 1.79] |
| Cardiovascular | 210 (55.4) | 35 (46.0) | 17 (47.6) | 0.77 [0.54, 1.10] | 0.75 [0.46, 1.23] | 0.97 [0.54, 1.73] |
| Heart failure-related | 127 (39.1) | 24 (35.2) | 9 (28.2) | 0.87 [0.57, 1.35] | 0.65 [0.33, 1.28] | 0.74 [0.34, 1.59] |
| Non-heart failure-related | 149 (42.8) | 17 (29.3) | 13 (38.8) | 0.52 [0.32, 0.87] | 0.82 [0.46, 1.44] | 1.51 [0.73, 3.11] |
| Non-cardiovascular | 66 (22.0) | 6 (15.4) | 5 (16.2) | 0.42 [0.18, 0.96] | 0.71 [0.28, 1.75] | 1.55 [0.47, 5.08] |
| Hospitalization for any cause | 386 (91.9) | 75 (90.1) | 35 (90.0) | 0.92 [0.72, 1.18] | 0.86 [0.61, 1.21] | 0.92 [0.62, 1.38] |
| Heart failure-related | 262 (71.3) | 55 (70.7) | 23 (69.8) | 0.95 [0.71, 1.26] | 0.87 [0.57, 1.33] | 0.91 [0.56, 1.47] |

Rates are time-to-first event Kaplan-Meier estimates presented as N events (%). Hazard ratios and 95% CIs are unadjusted values.

**Supplementary Table 9.** Clinical Outcomes Through 5-Year Follow-up by Race and Ethnicity and Treatment

|  | **White** | | | **Black** | | | **Hispanic** | | |  |
| --- | --- | --- | --- | --- | --- | --- | --- | --- | --- | --- |
|  | **M-TEER**  **(N=225)** | **GDMT Alone**  **(N=232)** | **HR (95% CI)** | **M-TEER**  **(N=44)** | **GDMT Alone**  **(N=44)** | **HR (95% CI)** | **M-TEER**  **(N=20)** | **GDMT Alone**  **(N=20)** | **HR (95% CI)** | **P_int_** |
| **Primary outcome:** | |  |  |  |  |  |  |  |  |  |
| All-cause mortality or HFH | 166 (76.2) | 196 (91.1) | 0.56 [0.46, 0.70] | 23 (59.4) | 39 (92.9) | 0.32 [0.19, 0.54] | 15 (77.5) | 17 (85.0) | 0.84 [0.42, 1.69] | 0.10 |
| All-cause mortality | 133 (61.9) | 143 (68.5) | 0.79 [0.62, 1.00] | 13 (36.7) | 28 (69.8) | 0.34 [0.17, 0.65] | 11 (56.4) | 11 (56.0) | 0.90 [0.39, 2.09] | 0.07 |
| First HFH | 113 (61.7) | 149 (82.2) | 0.52 [0.40, 0.66] | 20 (54.3) | 35 (86.3) | 0.32 [0.18, 0.57] | 11 (69.9) | 12 (70.6) | 0.86 [0.38, 1.96] | 0.16 |

Rates are Kaplan-Meier estimates presented as N events (%). GDMT = guideline-directed medical therapy; HFH = heart failure hospitalization; M-TEER = mitral transcatheter edge-to-edge repair.
